# Bis(2-hydroxy-*N*′-isopropylidenebenzo­hydrazidato-κ^2^ *N*′,*O*)bis­(pyridine-κ*N*)cobalt(II)

**DOI:** 10.1107/S1600536809011015

**Published:** 2009-03-28

**Authors:** Xiaojuan Zhao, Dacheng Li, Yupeng Pan

**Affiliations:** a College of Chemistry and Chemical Engineering, Liaocheng University, Shandong 252059, People’s Republic of China

## Abstract

In the title complex, [Co(C_10_H_11_N_2_O_2_)_2_(C_5_H_5_N)_2_], the Co^II^ atom lies on a centre of symmetry and adopts a distorted *cis*-CoO_2_N_4_ octa­hedral geometry. The two acetone salicyloylhydrazone ligands are deprotonated and act as *N*,*O*-bidentate monoanionic ligands, forming the equatorial plane, while the axial positions are occupied by two N atoms of two pyridine mol­ecules. The complex presents O—H⋯N and C—H⋯N intra­molecular hydrogen bonds. Inter­molecular C—H⋯N and C—H⋯O inter­actions are also present in the crystal.

## Related literature

For the crystal structure of acetone salicylhydrazone, see: Kraudelt *et al.* (1996 ▶). For the crystal structure of iron and nickel complexes with related aroylhydrazone derivatives, see: Matoga *et al.* (2007 ▶) and Liu *et al.* (2005 ▶), respectively. For the biological activity of aroylhydrazones, see: Armstrong *et al.* (2003 ▶). For the crystal structure of 3-hydr­oxy-*N*-[phen­yl(2-pyrid­yl)methyl­ene]-2-naphthohydrazide, see: Kang *et al.* (2007 ▶).
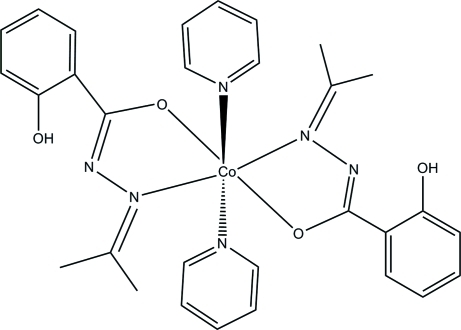


## Experimental

### 

#### Crystal data

[Co(C_10_H_11_N_2_O_2_)_2_(C_5_H_5_N)_2_]
*M* *_r_* = 599.55
Monoclinic, 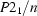

*a* = 7.7751 (9) Å
*b* = 10.0168 (15) Å
*c* = 18.751 (2) Å
β = 96.621 (2)°
*V* = 1450.6 (3) Å^3^
*Z* = 2
Mo *K*α radiation
μ = 0.64 mm^−1^
*T* = 298 K
0.34 × 0.19 × 0.16 mm

#### Data collection

Siemens SMART CCD area-detector diffractometer
Absorption correction: multi-scan (*SADABS*; Sheldrick, 1996 ▶) *T*_min_ = 0.813, *T*_max_ = 0.905
7087 measured reflections
2547 independent reflections
1675 reflections with *I* > 2σ(*I*)
*R*_int_ = 0.039

#### Refinement

*R*[*F* ^2^ > 2σ(*F* ^2^)] = 0.041
*wR*(*F* ^2^) = 0.106
*S* = 1.00
2547 reflections
187 parameters
H-atom parameters constrained
Δρ_max_ = 0.30 e Å^−3^
Δρ_min_ = −0.24 e Å^−3^

### 

Data collection: *SMART* (Siemens, 1996 ▶); cell refinement: *SAINT* (Siemens, 1996 ▶); data reduction: *SAINT*; program(s) used to solve structure: *SHELXS97* (Sheldrick, 2008 ▶); program(s) used to refine structure: *SHELXL97* (Sheldrick, 2008 ▶); molecular graphics: *SHELXTL* (Sheldrick, 2008 ▶); software used to prepare material for publication: *SHELXTL*.

## Supplementary Material

Crystal structure: contains datablocks I, global. DOI: 10.1107/S1600536809011015/bg2240sup1.cif

Structure factors: contains datablocks I. DOI: 10.1107/S1600536809011015/bg2240Isup2.hkl

Additional supplementary materials:  crystallographic information; 3D view; checkCIF report

## Figures and Tables

**Table 1:** Selected bond lengths (Å)

Co1—O1	2.028 (2)
Co1—O1^i^	2.028 (2)
Co1—N2^i^	2.179 (2)
Co1—N2	2.179 (2)
Co1—N3	2.233 (2)
Co1—N3^i^	2.233 (2)

Symmetry code: (i) 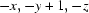.

**Table 2:** Hydrogen-bond geometry (Å, °)

*D*—H⋯*A*	*D*—H	H⋯*A*	*D*⋯*A*	*D*—H⋯*A*
O2—H2⋯N1	0.82	1.81	2.536 (3)	147
C11—H11⋯N2	0.93	2.56	3.157 (4)	123
C9—H9*A*⋯O1^i^	0.96	2.23	3.159 (4)	164
C15—H15⋯N2^i^	0.93	2.54	3.137 (4)	123

Symmetry code: (i) 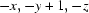.

**Fig. 1.**
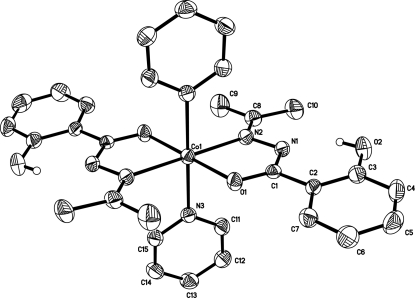
The molecular structure of the compound, showing 30% probability displacement ellipsoids. Unlabelled atoms are related to the labelled ones by symmetry operation (-x, 1 - y, -z). C-bound H atoms have been omitted for clarity.

## supplementary crystallographic information

### Comment

Aroylhydrazone and their metal complexes are of great importance owing to the wide spread applications in the fields of coordination chemistry and their biological activities. As an extension of our work on the structural characterization of aroylhydrazone derivatives (Liu *et al.*, 2005; Kang *et al.*, 2007), the title compound (I) was synthesized.

Fig. 1 shows a molecular view of (I). The complex consists of  one Co cation lying on a centre of symmetry [symmetry code: -*x*, 1 - *y*, -*z*], two acetone salicyloyl hydrazone ligands and two coordinated pyridine molecules. The monoanionic ligand (which is in its enol form, (C1—O1: 1.270 (3) Å) acts as bidentate forming the equatorial plane (Co1—O1(2.028 (2) Å and Co1—N2(2.179 (2) Å). Two pyridine molecules coordinate in the axial positions, the axial bond length (Co1—N3 2.233 (2) Å) being slightly longer than those of in the equatorial plane. In addition anumber of intramolecular (conventional) O—H···N and (non conventional) C—H···N, C—H···O H-bonds are found in the complex (Table 1).

### Experimental

To a stirred 10 ml pyridine solution of acetone salicyloylhydrazone (0.0384 g,0.2 mmol), 10 ml methanol solution of cobalt dichloride (0.0245 g,0.1 mmol) was added dropwise. The reaction mixture was stirred for 4 h at room temperature and then filtered. Brown single crystals were obtained from the filtrate after three weeks. Anal. Calcd (%) for C_30_H_32_O_4_N_6_Co (Mr = 599.55): C, 60.10; H, 5.38; N, 14.02; Found (%): C, 60.09; H, 5.38; N, 14.03

### Refinement

All H atoms were placed geometrically and treated as riding on their parent atoms with O—H 0.82 C—H 0.96 Å (methyl) [*U*_iso_(H) = 1.5*U*_eq_(C, O)] and C—H 0.93 (phenyl and pyridine) 0.93Å [*U*_iso_(H) = 1.2*U*_eq_(C)].

### Crystal data

**Table:**

[Co(C_10_H_11_N_2_O_2_)_2_(C_5_H_5_N)_2_]	*F*(000) = 626
*M**_r_* = 599.55	*D*_x_ = 1.373 Mg m^−^^3^
Monoclinic, *P*2_1_/*n*	Mo *K*α radiation, λ = 0.71073 Å
Hall symbol: -P 2yn	Cell parameters from 1792 reflections
*a* = 7.7751 (9) Å	θ = 2.3–21.6°
*b* = 10.0168 (15) Å	µ = 0.64 mm^−^^1^
*c* = 18.751 (2) Å	*T* = 298 K
β = 96.621 (2)°	Block, brown
*V* = 1450.6 (3) Å^3^	0.34 × 0.19 × 0.16 mm
*Z* = 2	

### Data collection

**Table:**

Siemens SMART CCD area-detector diffractometer	2547 independent reflections
Radiation source: fine-focus sealed tube	1675 reflections with *I* > 2σ(*I*)
graphite	*R*_int_ = 0.039
φ and ω scans	θ_max_ = 25.0°, θ_min_ = 2.2°
Absorption correction: multi-scan (*SADABS*; Sheldrick, 1996)	*h* = −9→9
*T*_min_ = 0.813, *T*_max_ = 0.905	*k* = −11→11
7087 measured reflections	*l* = −11→22

### Refinement

**Table:**

Refinement on *F*^2^	Primary atom site location: structure-invariant direct methods
Least-squares matrix: full	Secondary atom site location: difference Fourier map
*R*[*F*^2^ > 2σ(*F*^2^)] = 0.041	Hydrogen site location: inferred from neighbouring sites
*wR*(*F*^2^) = 0.106	H-atom parameters constrained
*S* = 1.00	*w* = 1/[σ^2^(*F*_o_^2^) + (0.046*P*)^2^ + 0.3767*P*] where *P* = (*F*_o_^2^ + 2*F*_c_^2^)/3
2547 reflections	(Δ/σ)_max_ < 0.001
187 parameters	Δρ_max_ = 0.30 e Å^−^^3^
0 restraints	Δρ_min_ = −0.23 e Å^−^^3^

### Special details

**Table:**

Geometry. All e.s.d.'s (except the e.s.d. in the dihedral angle between two l.s. planes) are estimated using the full covariance matrix. The cell e.s.d.'s are taken into account individually in the estimation of e.s.d.'s in distances, angles and torsion angles; correlations between e.s.d.'s in cell parameters are only used when they are defined by crystal symmetry. An approximate (isotropic) treatment of cell e.s.d.'s is used for estimating e.s.d.'s involving l.s. planes.
Refinement. Refinement of *F*^2^ against ALL reflections. The weighted *R*-factor *wR* and goodness of fit *S* are based on *F*^2^, conventional *R*-factors *R* are based on *F*, with *F* set to zero for negative *F*^2^. The threshold expression of *F*^2^ > σ(*F*^2^) is used only for calculating *R*-factors(gt) *etc*. and is not relevant to the choice of reflections for refinement. *R*-factors based on *F*^2^ are statistically about twice as large as those based on *F*, and *R*- factors based on ALL data will be even larger.

### Fractional atomic coordinates and isotropic or equivalent isotropic displacement parameters (Å^2^)

**Table:**

	*x*	*y*	*z*	*U*_iso_*/*U*_eq_	
Co1	0.0000	0.5000	0.0000	0.0385 (2)	
N1	0.0380 (3)	0.7734 (2)	0.06265 (13)	0.0414 (6)	
N2	0.1297 (3)	0.6915 (2)	0.01957 (13)	0.0423 (6)	
N3	0.1601 (3)	0.4115 (2)	0.09485 (13)	0.0437 (6)	
O1	−0.1480 (2)	0.59481 (19)	0.06648 (11)	0.0461 (5)	
O2	−0.0353 (3)	0.9926 (2)	0.11969 (14)	0.0671 (7)	
H2	0.0162	0.9415	0.0954	0.101*	
C1	−0.1001 (4)	0.7132 (3)	0.08301 (15)	0.0386 (7)	
C2	−0.2036 (4)	0.7937 (3)	0.12879 (16)	0.0413 (7)	
C3	−0.1665 (5)	0.9282 (3)	0.14443 (18)	0.0517 (9)	
C4	−0.2665 (5)	0.9984 (4)	0.1888 (2)	0.0663 (10)	
H4	−0.2436	1.0881	0.1985	0.080*	
C5	−0.3972 (6)	0.9365 (4)	0.2178 (2)	0.0767 (12)	
H5	−0.4619	0.9841	0.2479	0.092*	
C6	−0.4357 (5)	0.8035 (4)	0.2032 (2)	0.0748 (11)	
H6	−0.5254	0.7615	0.2233	0.090*	
C7	−0.3389 (4)	0.7348 (3)	0.15852 (18)	0.0564 (9)	
H7	−0.3655	0.6459	0.1480	0.068*	
C8	0.2667 (4)	0.7445 (3)	0.00010 (18)	0.0499 (8)	
C9	0.3747 (5)	0.6670 (4)	−0.0454 (2)	0.0804 (12)	
H9A	0.3273	0.5790	−0.0530	0.121*	
H9B	0.4907	0.6608	−0.0218	0.121*	
H9C	0.3760	0.7111	−0.0908	0.121*	
C10	0.3260 (5)	0.8822 (3)	0.0211 (2)	0.0697 (11)	
H10A	0.2458	0.9461	−0.0018	0.105*	
H10B	0.4386	0.8972	0.0064	0.105*	
H10C	0.3317	0.8918	0.0723	0.105*	
C11	0.2490 (4)	0.4881 (3)	0.14411 (17)	0.0553 (9)	
H11	0.2410	0.5803	0.1388	0.066*	
C12	0.3514 (4)	0.4381 (4)	0.20208 (19)	0.0621 (10)	
H12	0.4103	0.4957	0.2353	0.074*	
C13	0.3666 (4)	0.3029 (3)	0.21092 (19)	0.0620 (10)	
H13	0.4365	0.2669	0.2498	0.074*	
C14	0.2763 (4)	0.2223 (3)	0.16120 (18)	0.0593 (9)	
H14	0.2833	0.1299	0.1655	0.071*	
C15	0.1749 (4)	0.2803 (3)	0.10463 (18)	0.0520 (9)	
H15	0.1131	0.2245	0.0713	0.062*	

### Atomic displacement parameters (Å^2^)

**Table:**

	*U*^11^	*U*^22^	*U*^33^	*U*^12^	*U*^13^	*U*^23^
Co1	0.0379 (3)	0.0375 (3)	0.0410 (4)	−0.0085 (3)	0.0080 (2)	−0.0035 (3)
N1	0.0431 (15)	0.0393 (13)	0.0413 (15)	−0.0086 (12)	0.0032 (12)	−0.0028 (13)
N2	0.0413 (15)	0.0419 (14)	0.0440 (15)	−0.0102 (12)	0.0055 (12)	−0.0017 (12)
N3	0.0434 (15)	0.0448 (15)	0.0427 (16)	−0.0065 (12)	0.0039 (12)	0.0008 (13)
O1	0.0472 (12)	0.0390 (12)	0.0549 (14)	−0.0114 (10)	0.0177 (10)	−0.0065 (11)
O2	0.0715 (16)	0.0431 (13)	0.0864 (18)	−0.0074 (12)	0.0079 (14)	−0.0139 (13)
C1	0.0430 (18)	0.0383 (17)	0.0333 (17)	−0.0019 (14)	−0.0011 (14)	0.0028 (14)
C2	0.0469 (18)	0.0400 (17)	0.0357 (17)	0.0033 (14)	−0.0002 (14)	−0.0022 (15)
C3	0.057 (2)	0.049 (2)	0.046 (2)	0.0038 (17)	−0.0061 (17)	−0.0022 (17)
C4	0.081 (3)	0.052 (2)	0.064 (2)	0.016 (2)	0.000 (2)	−0.017 (2)
C5	0.086 (3)	0.086 (3)	0.060 (3)	0.034 (3)	0.016 (2)	−0.009 (2)
C6	0.081 (3)	0.070 (3)	0.079 (3)	0.015 (2)	0.035 (2)	0.005 (2)
C7	0.063 (2)	0.050 (2)	0.059 (2)	0.0054 (17)	0.0165 (18)	0.0016 (18)
C8	0.0446 (19)	0.051 (2)	0.054 (2)	−0.0164 (16)	0.0066 (16)	0.0036 (17)
C9	0.064 (2)	0.077 (3)	0.106 (3)	−0.024 (2)	0.035 (2)	−0.010 (3)
C10	0.064 (2)	0.057 (2)	0.089 (3)	−0.0276 (18)	0.011 (2)	0.001 (2)
C11	0.061 (2)	0.0479 (19)	0.053 (2)	−0.0063 (17)	−0.0081 (17)	−0.0033 (19)
C12	0.067 (2)	0.064 (2)	0.051 (2)	−0.0071 (19)	−0.0117 (19)	−0.0052 (19)
C13	0.065 (2)	0.066 (2)	0.052 (2)	−0.0037 (19)	−0.0074 (18)	0.009 (2)
C14	0.065 (2)	0.050 (2)	0.061 (2)	−0.0064 (17)	−0.0011 (19)	0.0112 (19)
C15	0.057 (2)	0.048 (2)	0.050 (2)	−0.0099 (16)	−0.0015 (17)	−0.0015 (17)

### Geometric parameters (Å, °)

**Table:**

Co1—O1	2.028 (2)	C5—H5	0.9300
Co1—O1^i^	2.028 (2)	C6—C7	1.374 (5)
Co1—N2^i^	2.179 (2)	C6—H6	0.9300
Co1—N2	2.179 (2)	C7—H7	0.9300
Co1—N3	2.233 (2)	C8—C9	1.483 (5)
Co1—N3^i^	2.233 (2)	C8—C10	1.492 (4)
N1—C1	1.326 (3)	C9—H9A	0.9600
N1—N2	1.403 (3)	C9—H9B	0.9600
N2—C8	1.280 (4)	C9—H9C	0.9600
N3—C15	1.330 (4)	C10—H10A	0.9600
N3—C11	1.332 (3)	C10—H10B	0.9600
O1—C1	1.270 (3)	C10—H10C	0.9600
O2—C3	1.335 (4)	C11—C12	1.366 (4)
O2—H2	0.8200	C11—H11	0.9300
C1—C2	1.481 (4)	C12—C13	1.368 (5)
C2—C7	1.379 (4)	C12—H12	0.9300
C2—C3	1.402 (4)	C13—C14	1.365 (4)
C3—C4	1.392 (5)	C13—H13	0.9300
C4—C5	1.357 (5)	C14—C15	1.375 (4)
C4—H4	0.9300	C14—H14	0.9300
C5—C6	1.386 (5)	C15—H15	0.9300
			
O1—Co1—O1^i^	180.00 (9)	C6—C5—H5	119.5
O1—Co1—N2^i^	103.38 (8)	C7—C6—C5	118.7 (4)
O1^i^—Co1—N2^i^	76.62 (8)	C7—C6—H6	120.6
O1—Co1—N2	76.62 (8)	C5—C6—H6	120.6
O1^i^—Co1—N2	103.38 (8)	C6—C7—C2	122.0 (3)
N2^i^—Co1—N2	180.00 (13)	C6—C7—H7	119.0
O1—Co1—N3	90.00 (9)	C2—C7—H7	119.0
O1^i^—Co1—N3	90.00 (9)	N2—C8—C9	119.4 (3)
N2^i^—Co1—N3	89.38 (9)	N2—C8—C10	123.4 (3)
N2—Co1—N3	90.62 (9)	C9—C8—C10	117.2 (3)
O1—Co1—N3^i^	90.00 (9)	C8—C9—H9A	109.5
O1^i^—Co1—N3^i^	90.00 (9)	C8—C9—H9B	109.5
N2^i^—Co1—N3^i^	90.62 (9)	H9A—C9—H9B	109.5
N2—Co1—N3^i^	89.38 (9)	C8—C9—H9C	109.5
N3—Co1—N3^i^	180.0	H9A—C9—H9C	109.5
C1—N1—N2	112.5 (2)	H9B—C9—H9C	109.5
C8—N2—N1	114.6 (2)	C8—C10—H10A	109.5
C8—N2—Co1	134.5 (2)	C8—C10—H10B	109.5
N1—N2—Co1	110.84 (16)	H10A—C10—H10B	109.5
C15—N3—C11	116.4 (3)	C8—C10—H10C	109.5
C15—N3—Co1	122.3 (2)	H10A—C10—H10C	109.5
C11—N3—Co1	121.4 (2)	H10B—C10—H10C	109.5
C1—O1—Co1	114.58 (18)	N3—C11—C12	123.3 (3)
C3—O2—H2	109.5	N3—C11—H11	118.4
O1—C1—N1	125.4 (3)	C12—C11—H11	118.4
O1—C1—C2	119.1 (3)	C11—C12—C13	119.6 (3)
N1—C1—C2	115.5 (3)	C11—C12—H12	120.2
C7—C2—C3	118.3 (3)	C13—C12—H12	120.2
C7—C2—C1	119.5 (3)	C14—C13—C12	118.2 (3)
C3—C2—C1	122.2 (3)	C14—C13—H13	120.9
O2—C3—C4	117.8 (3)	C12—C13—H13	120.9
O2—C3—C2	122.6 (3)	C13—C14—C15	118.7 (3)
C4—C3—C2	119.6 (4)	C13—C14—H14	120.6
C5—C4—C3	120.3 (4)	C15—C14—H14	120.6
C5—C4—H4	119.8	N3—C15—C14	123.8 (3)
C3—C4—H4	119.8	N3—C15—H15	118.1
C4—C5—C6	121.0 (4)	C14—C15—H15	118.1
C4—C5—H5	119.5		
			
C1—N1—N2—C8	−178.4 (3)	N1—C1—C2—C7	−173.4 (3)
C1—N1—N2—Co1	2.4 (3)	O1—C1—C2—C3	−174.8 (3)
O1—Co1—N2—C8	178.1 (3)	N1—C1—C2—C3	5.3 (4)
O1^i^—Co1—N2—C8	−1.9 (3)	C7—C2—C3—O2	178.2 (3)
N3—Co1—N2—C8	88.3 (3)	C1—C2—C3—O2	−0.5 (5)
N3^i^—Co1—N2—C8	−91.7 (3)	C7—C2—C3—C4	−0.3 (5)
O1—Co1—N2—N1	−2.81 (16)	C1—C2—C3—C4	−179.0 (3)
O1^i^—Co1—N2—N1	177.19 (16)	O2—C3—C4—C5	−177.3 (3)
N3—Co1—N2—N1	−92.66 (17)	C2—C3—C4—C5	1.2 (5)
N3^i^—Co1—N2—N1	87.34 (17)	C3—C4—C5—C6	−1.0 (6)
O1—Co1—N3—C15	120.1 (2)	C4—C5—C6—C7	−0.1 (6)
O1^i^—Co1—N3—C15	−59.9 (2)	C5—C6—C7—C2	1.0 (6)
N2^i^—Co1—N3—C15	16.7 (2)	C3—C2—C7—C6	−0.8 (5)
N2—Co1—N3—C15	−163.3 (2)	C1—C2—C7—C6	177.9 (3)
O1—Co1—N3—C11	−61.3 (2)	N1—N2—C8—C9	179.7 (3)
O1^i^—Co1—N3—C11	118.7 (2)	Co1—N2—C8—C9	−1.2 (5)
N2^i^—Co1—N3—C11	−164.6 (2)	N1—N2—C8—C10	0.0 (4)
N2—Co1—N3—C11	15.4 (2)	Co1—N2—C8—C10	179.1 (2)
N2^i^—Co1—O1—C1	−177.13 (19)	C15—N3—C11—C12	0.1 (5)
N2—Co1—O1—C1	2.87 (19)	Co1—N3—C11—C12	−178.6 (3)
N3—Co1—O1—C1	93.5 (2)	N3—C11—C12—C13	0.5 (6)
N3^i^—Co1—O1—C1	−86.5 (2)	C11—C12—C13—C14	−0.6 (6)
Co1—O1—C1—N1	−2.7 (4)	C12—C13—C14—C15	0.1 (5)
Co1—O1—C1—C2	177.38 (18)	C11—N3—C15—C14	−0.7 (5)
N2—N1—C1—O1	0.0 (4)	Co1—N3—C15—C14	178.0 (3)
N2—N1—C1—C2	180.0 (2)	C13—C14—C15—N3	0.6 (5)
O1—C1—C2—C7	6.5 (4)		

Symmetry codes: (i) −*x*, −*y*+1, −*z*.

### Hydrogen-bond geometry (Å, °)

**Table:**

*D*—H···*A*	*D*—H	H···*A*	*D*···*A*	*D*—H···*A*
O2—H2···N1	0.82	1.81	2.536 (3)	147
C7—H7···O1	0.93	2.46	2.782 (4)	100
C11—H11···N2	0.93	2.56	3.157 (4)	123
C9—H9A···O1^i^	0.96	2.23	3.159 (4)	164
C15—H15···N2^i^	0.93	2.54	3.137 (4)	123

Symmetry codes: (i) −*x*, −*y*+1, −*z*.
